# Genetic and Behavioral Investigations into Odor Discrimination and Memory

**DOI:** 10.1371/journal.pbio.0030388

**Published:** 2005-10-18

The smell of baking bread, the perfume of flowers, the tang of sea air—your nose can sense and distinguish between these smells and thousands more. The sense of smell—managed by the olfactory system—is a crucial tool for sensing the environment. Thousands of low molecular weight molecules bind to a vast repertoire of odor receptors on olfactory sensory neurons in the nose. These neurons extend long projections into an area of the forebrain known as the olfactory bulb, where chemical messengers (neurotransmitters) pass on information to other neurons elsewhere in the brain. In ways that are only just beginning to be understood, all this information is integrated by neural circuits in the brain so that different odors can be learned and discriminated; in addition, changes in neuron activity are responsible for remembering odors.

The neural circuits that underlie odor learning and discrimination and olfactory memory rely on neurotransmission that is mediated by ion channels (pores that allow ions to pass through the normally impermeable cell membrane) called γ-amino-3–hydroxy-5–methyl-4–isoxazoleproprionate receptors (AMPARs). Each AMPAR is comprised of multiple subunits of glutamate receptors (GluRs), which form the ion channels. Most AMPARs contain GluR-B, which controls many of their properties, including their permeability to calcium ions.

Derya Shimshek, Andreas Schaefer, and their colleagues are combining genetic studies and quantitative behavioral studies to assess how AMPARs contribute to olfactory learning, discrimination, and memory. To investigate these processes, the researchers have constructed two sets of genetically modified mice. In the first set, some neurons in the forebrain express a form of GluR-B that increases the calcium ion permeability of AMPARs. In the second set, GluR-B expression is partially reduced in the forebrain, a manipulation that also renders AMPARs calcium permeable.

In behavioral tests, in which mice were rewarded for their ability to discriminate between similar test odors, genetically altered mice showed quicker olfactory learning and increased discriminatory prowess than mice without genetic alterations in GluR-B. Thus, increased AMPAR-mediated entry of calcium ions into neurons within the forebrain, in particular within the olfactory bulb, seems to enhance olfactory learning and discrimination. By contrast, GluR-B-depleted mice showed impaired odor memory. Different GluR-B-depleted mice had different degrees of memory impairment (even though they all had similar improvements in odor learning and discrimination). This variability, the researchers suggest, could reflect regional differences in the expression of residual GluR-B produced by the genetic manipulations used to derive the mice. When the scientists investigated this idea by measuring GluR-B expression in various areas of the brains of mice whose behavior they had already tested, they found that the decreases in olfactory memory in individual GluR-B-depleted mice strongly correlated with reductions in GluR-B levels in their hippocampus and piriform cortex. Taken together with data from other groups that discounts the involvement of the hippocampus in the type of long-term olfactory memory tested here, this result strongly supports the prevalent view that the piriform cortex is important in olfactory memory. [Fig pbio-0030388-g001]

**Figure pbio-0030388-g001:**
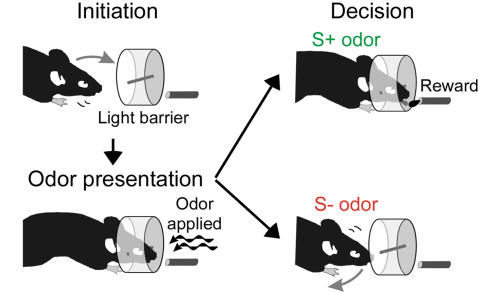
Water-deprived mice are trained to distinguish a water-rewarded odor (S+) and an unrewarded odor (S−) by their licking response

Overall, the results presented by Shimsek and colleagues suggest that olfactory discrimination and memory in mice are achieved in mechanistically and spatially distinct ways even though the same ion channel receptor is involved. The researchers' experimental approach—combining quantitative behavioral analyses with genetic manipulations that introduce variable patterns of gene expression into the brain—should prove invaluable for dissecting the neural circuitry underlying not only olfaction but also other sensory systems in mice. And because these systems are very highly conserved, finding out about mouse olfaction should also indicate how humans sniff out good and bad smells. —*Jane Bradbury*

